# Entropy Generation and Thermoelastic Damping in the In-plane Vibration of Microring Resonators

**DOI:** 10.3390/e21070631

**Published:** 2019-06-27

**Authors:** Yongpeng Tai, Pu Li, Yan Zheng, Jie Tian

**Affiliations:** 1 College of Automobile and Traffic Engineering, Nanjing Forestry University, Nanjing 210037, China; 2 School of Mechanical Engineering, Southeast University, Nanjing 211189, China

**Keywords:** thermoelastic damping, entropy generation, quality factor, ring resonator, in-plane mode

## Abstract

Thermoelastic damping is a critical issue for designing very high quality factor microresonators. This paper derives the entropy generation, associated with the irreversibility in heat conduction, that is used for ring resonators in in-plane vibration and presents an analytical model of thermoelastic damping according to heat increments calculated by entropy theory. We consider the heat flow only in radial thickness of the ring and obtain a complex temperature field that is out of phase with the mechanical stress. The thermoelastic dissipation is calculated in the perspective of heat increments that appear due to entropy generation. The analytical model is validated by comparing with an LR (Lifshitz and Roukes) model, finite-element method and measurement. The accuracy of the present model is found to be very high for different ambient temperatures and structures. The effects of structure dimensions and vibration frequencies on entropy generation and thermoelastic damping is investigated for ring resonators under in-plane vibration.

## 1. Introduction

Microrings are a common resonator of MEMS (a microelectromechanical system), which are widely applied to MEMS sensors and actuators [1,2,3,4]. The ring resonators are usually operating in two kinds of vibrating mode, i.e., in-plane mode and out-of-plane mode. The performance of a microresonator is closely related to the quality factor, which is the critical issue for the sensitivity and resolution of MEMS systems. A high quality factor indicates low energy consumption. In order to design very high quality factor microresonators, it is necessary to study the prevailing energy dissipation mechanisms in MEMS. Among these mechanisms, air damping and clamping losses are the main extrinsic losses that can be minimized by vacuum environment and optimal geometric design. Thermoelastic Damping (TED) is a dominant source of intrinsic loss in MEMS that cannot be completely eliminated by improved design or fabrication compared to extrinsic losses. Accordingly, thermoelastic damping usually plays an important role for designing high quality factor microresonators.

In a vibrating structure, there is a mutual coupling between the strain field and temperature field which generates irreversible heat conduction across the oscillating temperature gradient. It means that mechanical energy is transformed into heat with the generation of entropy. This kind of energy dissipation mechanism is referred to as TED. The theory of TED was first studied by Zener [5,6]. He proposed an accurate analytical expression of TED in beam vibrations by using the temperature field in the form of thermal modes. In 2000, Lifshitz and Roukes (LR) [7] presented a refined theory of thermoelastic damping using the same fundamental physics of Zener theory, in which a complex temperature field is obtained instead of thermal modes temperature field. Nowadays, the two models are widely used in MEMS resonators.

Thermoelastic damping is also studied in various structures of microresonators, such as a beam [8], ring [9], plate [10], shell [11], and so on. Zener and LR theories are applicable to beam resonators in flexure vibration [7]. An analytical model of TED in microplates has also been investigated by using the similar analysis of Zener and LR theories [10,12,13,14,15]. The ring resonator is a flexural beamlike geometry under in-plane vibration. Thus, its analytical model of TED is similar to the Zener and LR models [9,16,17,18,19]. Wong et al [9] studied TED for in-plane ring resonators based on the LR theory and presented an analytical model, which is similar to the LR model of beams. However, the characteristic length in the analytical model for a ring is the radial thickness, which is different from the beam depth in LR model. Kim et al [20] studied the effect of geometrical imperfections on the TED of ring resonators. The out-of-plane mode of a ring is a coupled motion that is affected by the coupling of flexure and torsion. It is different from an in-plane mode and is more difficult to use to predict TED. However, the analytical solution of TED in the out-of-plane mode of a ring has also been studied [21].

Thermoelastic damping can be obtained from two different ways, mechanical work loss and entropy generation. Zener [6] verified the equivalence of the mechanical work loss method and the entropy generation method. To date, the usual method to derive analytical models of TED in microresonators is calculating the mechanical work loss [22]. However, the entropy generation method is also used to evaluate TED in MEMS devices [13,22,23,24,25]. In previous work [22], we presented analytical models of TED for flexural vibration resonators including microbeams and microplates by using the entropy generation method. These models were proven to be valid and accurate, and so can be used as an alternative to other theories.

Other than the aforementioned review, few works study the analytical approximation of TED in a ring resonator from the perspective of entropy generation. In this paper, the entropy generation will be investigated for ring resonators of in-plane vibration. Using a complex temperature field and entropy generation method, we will present a simple analytical expression for TED in a ring resonator, which is different from the expressions of Zener and LR models. Compared to Zener theory, the present model uses a complex temperature field to calculate the entropy generation and heat increments instead of using a modal superposition temperature field to calculate mechanical work loss in which the first thermal mode is usually dominant and reserved. The validation of the analytical model in this paper will be confirmed by comparing with LR theory, FEM (finite-element method) and measurement. We will also study the characteristics of entropy generation and thermoelastic damping for a ring resonator under in-plane vibration.

The analytical model in this paper is not only different from the model of Wong et al [9] calculated by mechanical energy loss based on the LR theory but is also different from the Zener theory. It can be an alternative method with high accuracy to predict TED in ring resonators and provide verification from the perspective of entropy generation for other methods. This paper derives a specific expression of entropy generation in ring resonators of in-plane mode, which depends on the radial displacement. The simplification of this paper is different from Zener and LR theories. The Zener method usually ignores high-order thermal modes. The LR frequency attenuation method simplifies the complex resonant frequency; the LR energy method neglects thermal stress when calculating mechanical energy loss. The entropy generation method simplifies the temperature using Taylor expansion. The method used does not need to consider the frequency attenuation and thermal strain of mechanical vibration when calculating the entropy production and energy loss. It only needs to know the temperature field function. This solution technology can improve the computational efficiency by avoiding the complex frequency [26].

## 2. Problem Formulation

From the second law of thermodynamics, entropy generation results from the irreversible heat flow caused by the compression and tension of an oscillating structure. This irreversible process of heat conduction results in a conversion of useful mechanical energy into heat and hence, causes thermoelastic damping [24]. For vibrating resonators during per cycle, the energy lost is the mechanical work loss ∆*W* as well as the heat increase Δ*Q* related to entropy [6]. The relationship between the mechanical work lost, heat increment and entropy generation can be expressed as

(1) $$\Delta W=\Delta Q=T_{0}\Delta S$$

where *T*_0_ is the ambient temperature, ∆*S* is the generation of entropy. Consequently, the TED can be obtained by using the definition of quality factor, given by

(2) $$Q^{-1}=\frac{1}{2\pi }\frac{\Delta W}{W_{\mathrm{stored}}}$$

where *W*_stored_ is the maximum energy stored per cycle. This is the entropy generation method to estimate TED.

### 2.1. Heat Conduction Governing Equation

**Figure 1:**
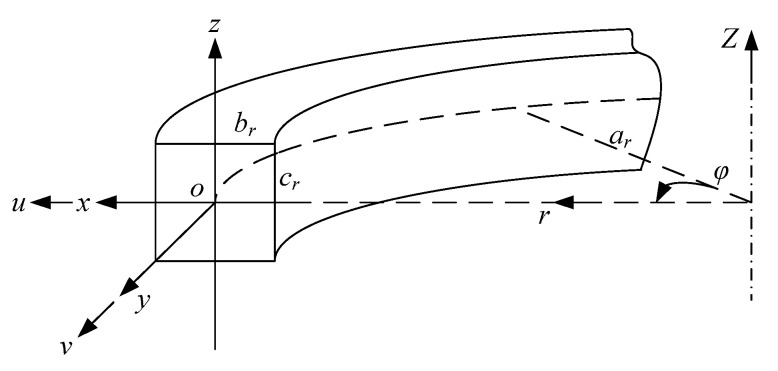
Schematic diagram of a ring resonator with coordinate systems.

We consider a free boundary ring resonator of rectangular cross-section, Figure 1, with the mean radius *a_r_*, the radial thickness *b_r_* and the axial width *c_r_*. A global cylindrical coordinate system (*r*, *φ*, *Z*) and a local Cartesian coordinate system (*x*, *y*, *z*) are defined. *u*, *v* and *w* denote the radial displacement, tangential displacement and translation displacement, respectively. Silicon rings as a common element in MEMS device are capable of both in-plane and out-of-plane flexural vibration. We study only the in-plane vibration in this paper.

**Figure 2:**
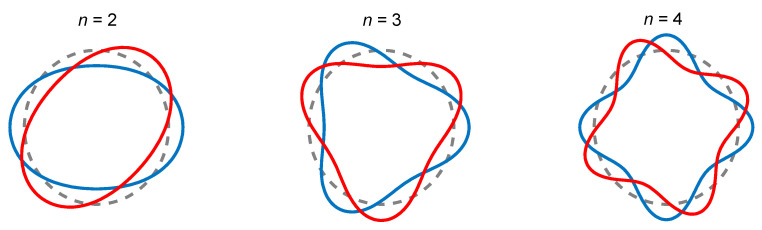
In-plane modes of a ring resonator with mode number *n* = 2, 3 and 4. The two lines of red and blue in each mode represent an equivalent degenerate pair with a mutual angle of *π*/2*n*.

Figure 2 shows the in-plane modes of a ring with free boundary conditions. The displacements of a ring under in-plane vibration can be expressed as [27]

(3) $$\left\{ \begin{array}{l} u\left(\varphi ,t\right)=U\left(\varphi \right)e^{i\omega t}=U_{0}\cos\left(n\varphi \right)e^{i\omega t}\\ v\left(\varphi ,t\right)=V\left(\varphi \right)e^{i\omega t}=V_{0}\sin\left(n\varphi \right)e^{i\omega t} \end{array}\right.$$

where *n* = 2, 3, 4… is the mode number, *ω* is the eigenfrequency corresponding to in-plane mode of a ring, *U* and *V* are the amplitudes of the ring in radial and tangential displacements, respectively, and *U*_0_ and *V*_0_ are the maximums of *U* and *V*. For low-order modes of rings, the relationship between *U*_0_ and *V*_0_ can be expressed as [27]

(4) $$\frac{U_{0}}{V_{0}}=-n$$

Considering thermoelastic effects, the strain fields of the ring can be expressed as [17]

(5) $$\left\{ \begin{array}{l} \varepsilon_{\varphi }=\frac{\sigma_{\varphi }}{E}+\alpha \theta \\ \varepsilon_{r}=\varepsilon_{Z}=-\frac{\upsilon }{E}\sigma_{\varphi }+\alpha \theta \end{array}\right.$$

where *ε_φ_*, *ε_r_* and *ε_Z_* are the circumferential, radial and axial strains in the ring, respectively, *σ_φ_* is the circumferential stress, *E* is the Young’s modulus, *α* is the coefficient of thermal expansion, and *θ* is the temperature change with respect to the ambient temperature *T*_0_, given by

(6) $$\theta \left(r,\varphi ,Z,t\right)=\theta_{0}\left(r,\varphi ,Z\right)e^{i\omega t}$$

For small vibration, the strain and stress along circumferential direction can be obtained by using the radial displacement *u*, given by [17]

(7) $$\varepsilon_{\varphi }=-\frac{x}{a_{r}^{2}}\left(\frac{\partial^{2}u}{\partial \varphi^{2}}+u\right)$$

(8) $$\sigma_{\varphi }=-\frac{Ex}{a_{r}^{2}}\left(\frac{\partial^{2}u}{\partial \varphi^{2}}+u\right)-E\alpha \theta .$$

where *x* is the local coordinate of the ring. The in-plane mode of a ring resonator is accompanied by internal heat conduction. According to the Fourier Law, the governing equation for heat conduction is given by [28]

(9) $$\frac{\partial \theta }{\partial t}=\chi \nabla^{2}\theta -\frac{E\alpha T}{\left(1-2\upsilon \right)C_{v}}\frac{\partial e_{\mathrm{cubic}}}{\partial t},$$

where ∇^2^(*r*, *φ*, *Z*) is the Laplacian operator in Cylindrical coordinate system, *χ* is the thermal diffusivity, *υ* is Poisson’s ratio, *C_v_* is the heat capacity per unit volume, and *e*_cubic_ = *ε_φ_* + *ε_r_* + *ε_Z_* is the cubic dilation. The Laplacian operator can be expressed as

(10) $$\nabla^{2}=\frac{\partial^{2}}{\partial r^{2}}+\frac{1}{r}\frac{\partial }{\partial r}+\frac{1}{r^{2}}\frac{\partial^{2}}{\partial \varphi^{2}}+\frac{\partial^{2}}{\partial Z^{2}}.$$

Substituting Equation (5) into Equation (9), yields

(11) $$\left(1+2\Delta_{E}\frac{1+\upsilon }{1-2\upsilon }\right)\frac{\partial \theta }{\partial t}=\chi \nabla^{2}\theta +\frac{\Delta_{E}}{\alpha }\frac{\partial }{\partial t}\left[\frac{x}{a_{r}^{2}}\left(\frac{\partial^{2}u}{\partial \varphi^{2}}+u\right)\right],$$

where Δ*_E_* = *Eα*^2^*T*_0_/*C_v_* is relaxation strength of the Young’s modulus.

To simplify, several assumptions are made for the governing equation. First, replace *T* by *T*_0_ due to the fact that *θ* << *T*_0_, and neglect the heat conduction in circumferential and axial directions (along *y* and *z* axes) [29]. Because the eigenfrequency of in-plane mode is high, the heat conduction in the circumferential state can be neglected due to long heat transfer distance and large relaxation time. Second, ignore small amounts, e.g., 2∆*_E_*(1 + *υ*)/(1 − 2*υ*), and remove the nonlinearity from the problem. Thus, the reduced heat equation along the *x*-direction can be expressed as [9]

(12) $$\frac{\partial \theta }{\partial t}=\chi \frac{\partial^{2}\theta }{\partial x^{2}}+\frac{\Delta_{E}}{\alpha }\frac{\partial }{\partial t}\left[\frac{x}{a_{r}^{2}}\left(\frac{\partial^{2}u}{\partial \varphi^{2}}+u\right)\right]$$

Typically, the above equation is one-way coupled so that the temperature field does not affect the stress field, because the thermal stresses are negligibly small compared to the mechanical stresses.

Assuming that the surfaces of the ring is adiabatic, then the boundary conditions are ∂*θ*/∂*x* = 0 at *x* = ± *b_r_*/2. Using heat conduction governing Equation (12), the temperature profile for a ring is given by [9]

(13) $$\theta_{0}\left(x,\varphi \right)=\frac{\Delta_{E}}{\alpha }\frac{1}{a_{r}^{2}}\left(\frac{\partial^{2}U\left(\varphi \right)}{\partial \varphi^{2}}+U\left(\varphi \right)\right)\left[x-\frac{\sin\left(px\right)}{p\cos\left(\frac{pb_{r}}{2}\right)}\right],$$

where

(14) $$p=\left(1-i\right)\sqrt{\frac{\omega }{2\chi }}.$$

### 2.2. Entropy Generation

In the discussion that follows, the entropy generation in system is derived based on the second law of thermodynamics. For convenience, we rewrite the temperature field,

(15) $$\theta \left(r,\varphi ,Z,t\right)=\theta_{0}\left(r,\varphi ,Z\right)\sin\left(\omega t\right)$$

The rate of entropy generation per unit volume can be expressed as [25]

(16) $${\dot{s}}_{g}=\kappa \frac{\nabla \theta \cdot \nabla \theta }{T^{2}}$$

Substituting Equation (15) into Equation (16) yields

(17) $${\dot{s}}_{g}=\frac{\kappa }{T^{2}}{\left(\frac{\partial \theta_{0}}{\partial x}\right)}^{2}\sin^{2}\left(\omega t\right)$$

In Equation (17), we consider the temperature gradient only in the *r*-direction. To avoid the nonlinear problem in Equation (17), we expand *T*^−2^ in Taylor series up to the first order,

(18) $$\frac{1}{T^{2}}=\frac{1}{T_{0}^{2}}-\frac{2}{T_{0}^{3}}\theta$$

Using Equations (17) and (18), the entropy generation per unit volume over a cycle is

(19) $$\Delta s=\int {\dot{s}}_{g}dt=\frac{\pi \kappa }{\omega T_{0}^{2}}{\left\{\frac{\Delta_{E}}{\alpha }\frac{1}{a_{r}^{2}}\left(\frac{\partial^{2}U\left(\varphi \right)}{\partial \varphi^{2}}+U\left(\varphi \right)\right)\right\}}^{2}{\left(1-\frac{\cos\left(px\right)}{\cos\left(\frac{pb_{r}}{2}\right)}\right)}^{2}$$

For a ring resonator, the total entropy generation per cycle is (see Appendix for detail)

(20) $$\Delta S=\int_{V}\Delta sdV=\frac{A_{r}}{\xi^{2}}\cdot \frac{\Delta_{E}Ec_{r}\pi^{2}{\left(n^{2}+1\right)}^{2}U_{0}^{2}b_{r}^{3}}{2T_{0}a_{r}^{4}},$$

where $\xi =b_{r}\sqrt{\left(\omega /2\chi \right)}$ is a dimensionless variable and *A_r_* is a coefficient, given by

(21) $$A_{r}=1+\frac{1}{1+\cos\left(pb_{r}\right)}-\frac{3}{pb_{r}}\tan\left(\frac{pb_{r}}{2}\right)$$

Note that *A_r_* is a complex number and *pb_r_* = (1 − *i*)*ξ*. The modulus of *A_r_* can be expressed as

(22) |Ar|=Re2(Ar)+Im2(Ar)

where the real and imaginary parts of *A_r_* are given by

(23) Re(A)=1+1+coshξcosξ(coshξ+cosξ)2−32ξsinhξ+sinξcoshξ+cosξ

(24) Im(A)=32ξsinhξ−sinξcoshξ+cosξ−sinhξsinξ(coshξ+cosξ)2

Accordingly, the modulus of entropy generation can be expressed as

(25) $$\left|\Delta S\right|=\frac{\left|A_{r}\right|}{\xi^{2}}\cdot \frac{\Delta_{E}Ec_{r}\pi^{2}{\left(n^{2}+1\right)}^{2}U_{0}^{2}b_{r}^{3}}{2T_{0}a_{r}^{4}}$$

Note that for a ring resonator with specific structure dimensions, the entropy generation depends only on the maximum of the radial displacement *U*_0_, $\Delta S\propto U_{0}^{2}$, which is related to the magnitude of the exciting force. 

### 2.3. Thermoelastic Damping

According to Equation (1), the heat content increment is equal to mechanical energy loss. Thus, the total work loss of the entire ring per cycle is given by

(26) $$\begin{array}{cc} \Delta W & =T_{0}\cdot \left|\Delta S\right|\\ & =\frac{\left|A_{r}\right|}{\xi^{2}}\cdot \frac{\Delta_{E}Ec_{r}\pi^{2}{\left(n^{2}+1\right)}^{2}U_{0}^{2}b_{r}^{3}}{2a_{r}^{4}} \end{array}$$

Using Equations (7) and (8), the maximum energy stored of a ring per cycle of in-plane vibration can be obtained as (see Appendix for detail)

(27) $$W_{\mathrm{stored}}=\frac{1}{2}\int_{V}{\hat{\sigma }}_{\varphi }{\hat{\varepsilon }}_{\varphi }dV=\frac{Ec_{r}\pi {\left(n^{2}+1\right)}^{2}U_{0}^{2}b_{r}^{3}}{24a_{r}^{4}}$$

Here, the thermoelastic component of *σ_φ_* is usually omitted due to negligible effect. 

Substituting Equations (26) and (27) into Equation (2), we obtain the analytical expression for thermoelastic damping of a ring, given by

(28) $$\begin{array}{cc} Q_{\mathrm{ring}}^{-1} & =\frac{6\Delta_{E}}{\xi^{2}}\{{\left[1-\frac{3}{2\xi }\frac{\sin\xi +\sinh\xi }{\cos\xi +\cosh\xi }+\frac{1+\cos\xi \cosh\xi }{{\left(\cos\xi +\cosh\xi \right)}^{2}}\right]}^{2}\\ & {+{\left[\frac{3}{2\xi }\frac{\sinh\xi -\sin\xi }{\cos\xi +\cosh\xi }-\frac{\sin\xi \sinh\xi }{{\left(\cos\xi +\cosh\xi \right)}^{2}}\right]}^{2}\}}^{\frac{1}{2}} \end{array}$$

Note that this analytical model for a ring is exactly the same as that for a rectangular beam in Ref. [22], except that the beam thickness is replaced by the radial thickness of the ring. Table 1 lists the comparison of the Zener model, LR model and the present model. It should be noted that the three models have the same scope of application.

## 3. Results and Discussions

In this section, first the thermoelastic damping model of this paper for ring resonators is validated by comparing with the experimental data and other theories results. Second, the geometry effect on TED is studied by using the present method and LR method. Third, the dependence of entropy generation on mode numbers and structure dimensions is discussed. The material properties of the ring resonator are from Table 2 unless otherwise specified.

### 3.1. Verification of the Present Model

First, we compare the present model with experimental data. Wong et al. [18] gave experimental measurements of TED for a practically relevant range of ring sizes. The following material properties are used in making the theoretical predictions: *α* = 2.6 × 10^−6^ K^−1^, *E* = 165 GPa, *C_v_* = 1.64 × 10^−6^ J m^−3^ K^−1^, *ρ* = 2330 kg m^−3^, *κ* = 147 W m^−1^ K^−1^, *χ* = 8.6 × 10^−5^ m^2^ s^−1^. Table 3 shows a comparison of measured and predicted TED in rings with different dimensions. The resonators are operating at the mode *n* = 2. Note that the values predicted by the present model are in good agreement with the experimental data. The maximum difference is of the order of 10% for all cases.

**Figure 3:**
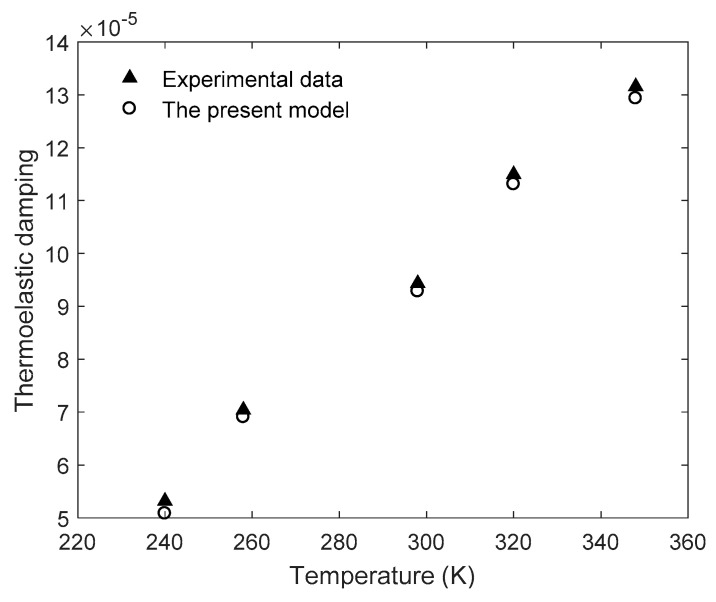
Variation of thermoelastic damping with temperatures in a silicon ring resonator.

Wong et al [18] also studied the experimental measurements of TED for different temperatures. Figure 3 shows the variation of TED with temperatures in a silicon ring resonator. The silicon ring resonator is operating at the mode of *n* = 2 (13.8 kHz) with critical dimensions of *a_r_* = 3 mm and *b_r_* = 120 μm. The material properties of silicon at various temperatures are used from the literature [18] (listed in Table 4). The material properties vary with temperatures and we use five sets of material properties associated with five different temperatures to calculate TED. From Figure 3, the results of the present model based on entropy are very close to the measurements, and the maximum difference is within 5% for all cases.

**Figure 4:**
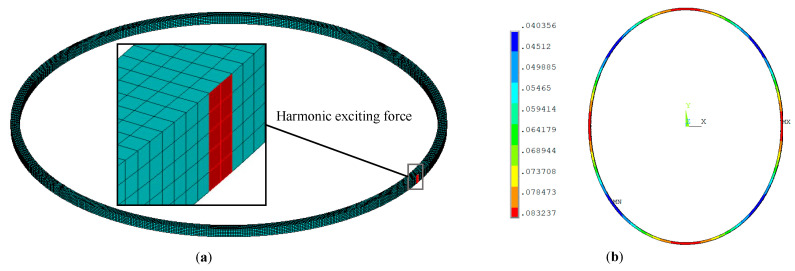
The simulation of FEM for a ring of in-plane vibration with free boundary conditions. (**a**) Meshing graph of FEM for a ring with exciting force applied on the surface. (**b**) The results of displacement at the fundamental mode *n* = 2 of in-plane vibration.

**Figure 5:**
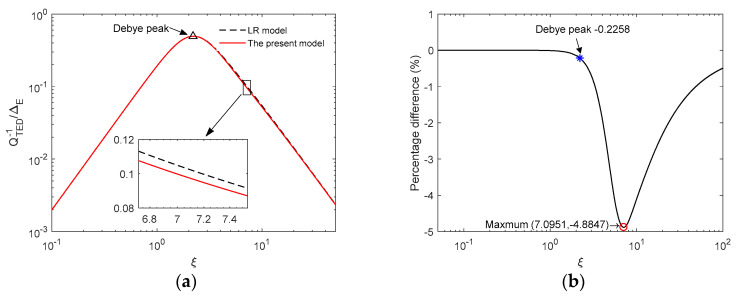
Comparison between LR model and the present model for ring resonators. (**a**) The normalized thermoelastic damping curves. (**b**) Percentage difference of thermoelastic damping between LR model and the present model.

**Figure 6:**
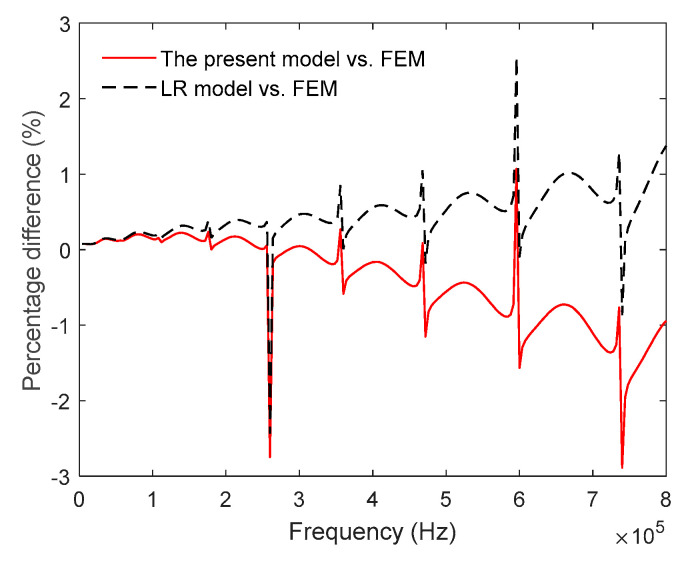
Percentage difference of thermoelastic damping between analytical models and FEM.

Next, we compare the present model with LR theory and FEM. Wong et al [9] investigated thermoelastic damping in in-plane vibration of ring resonators based on the LR theory and presented an analytical model which is the same as LR model of beams, except that the characteristic length is the radial thickness of the ring instead of the depth of the beam. The analytical expression is used for comparison in this section. The FEM results are obtained by ANSYS with element type SOLID226. In the simulation, the boundary conditions of the ring are free and the exciting force is applied on a small surface of the ring (shown in Figure 4a). Using harmonic analysis, we solve the three-dimensional thermo-mechanical equations, obtain the total strain energy of all elements under harmonic excitation in the range of 4 kHz ~ 800 kHz and then predict TED by calculating the ratio of the imaginary part to the real part of the total strain energy. Figure 4b shows the displacement of the ring obtained by FEM at the in-plane mode *n* = 2. From the figure, it illustrates that the vibration displacement of a ring simulated by FEM is consistent with the vibration theory. Figure 5 and Figure 6 show the comparisons of TED calculated by the LR model, FEM and the present model, respectively. As shown in Figure 5a, the results of the present model and the LR method are in good agreement with each other. At high frequency, the prediction of the present model is slightly smaller than that of the LR method. From Figure 5b, the maximum difference between the LR method and the present model is within 5%, which occurs at *ξ* ≈ 7.1. Note that the difference at Debye peak is about 0.2%. Figure 6 shows the relative error (in percent) of the results between the FEM and the present model for the case of *a_r_* = 1 mm and *b_r_* = *c_r_* = 20 μm. As shown in Figure 6, the predictions of the present model differ for FEM simulation by less than 3% depending on vibration frequency. Note that the predictions of the present model are basically smaller than the simulation results of FEM. Also shown in Figure 6, the LR method is used for comparison, and its results are larger than the FEM simulation.

### 3.2. Geometry effect on TED

For ring resonators, the structure dimensions can affect the eigenfrequency as well as TED, which strongly depends on the vibrating frequency. We study the effect of geometry on TED in polysilicon ring resonators by using the present model based on entropy theory. Theoretically, the in-plane mode and TED are independent of the axial width *c_r_*, and hence, we do not consider the effect of *c_r_* in this section.

**Figure 7:**
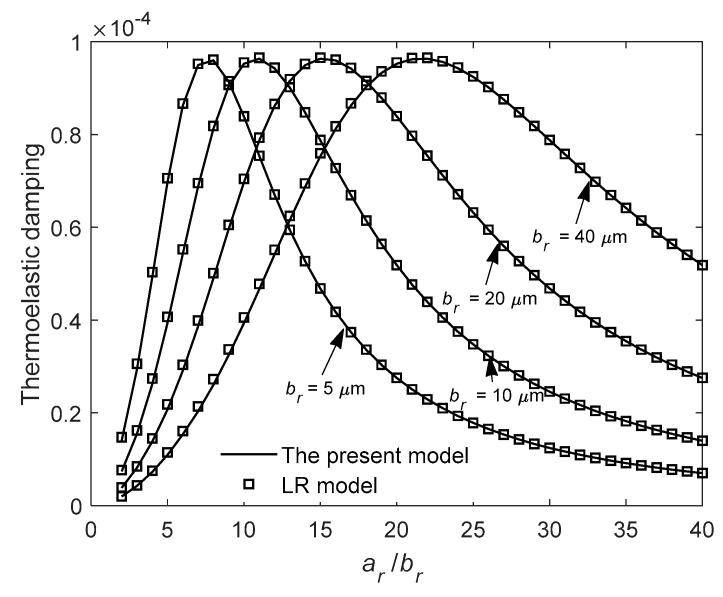
Variation of thermoelastic damping with the ratio *a_r_*/*b_r_* for different radial thickness *b_r_* at *n* = 2.

**Figure 8:**
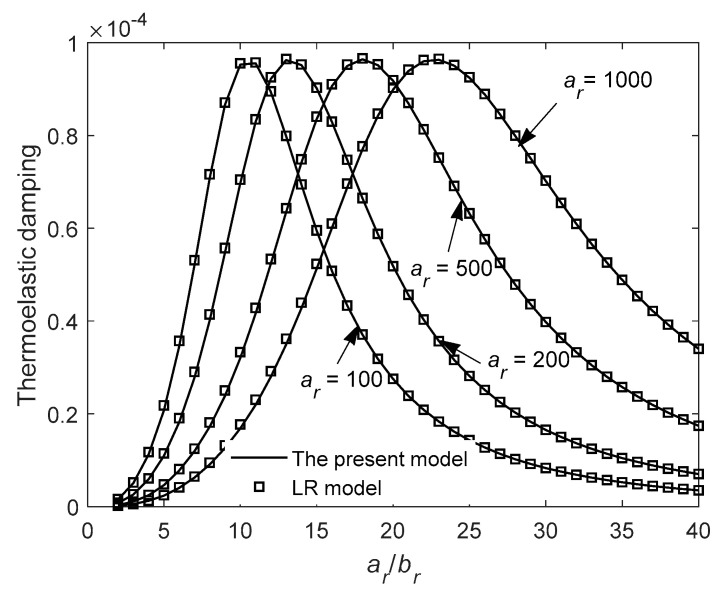
Variation of thermoelastic damping with the ratio *a_r_*/*b_r_* for different mean radius *a_r_* at *n* = 2.

We study the relationship between TED and the ratio *a_r_*/*b_r_* at mode *n* = 2 for the case of *b_r_* = *c_r_*. Figure 7 shows variation of TED of the first in-plane mode with *a_r_*/*b_r_* for different radial thickness *b_r_*. As shown in Figure 7, a peak value of TED exists as the ratio *a_r_*/*b_r_* changes. Note that for a larger radial thickness *b_r_*, a larger *a_r_*/*b_r_* exists that corresponds to the peak value of TED. Figure 8 shows variation of TED of the first in-plane mode with *a_r_*/*b_r_* for different mean radius *a_r_*. As shown in Figure 8, increasing mean radius *a_r_* results in increasing *a_r_*/*b_r_* associated with the peak value. From Figure 7 and Figure 8, the peak value is constant and does not change with the structure dimensions *a_r_* or *b_r_*. This characteristic will greatly reduce the difficulty of structural optimization design of ring resonators. The results of LR model are also plotted for comparison in the figures.

**Figure 9:**
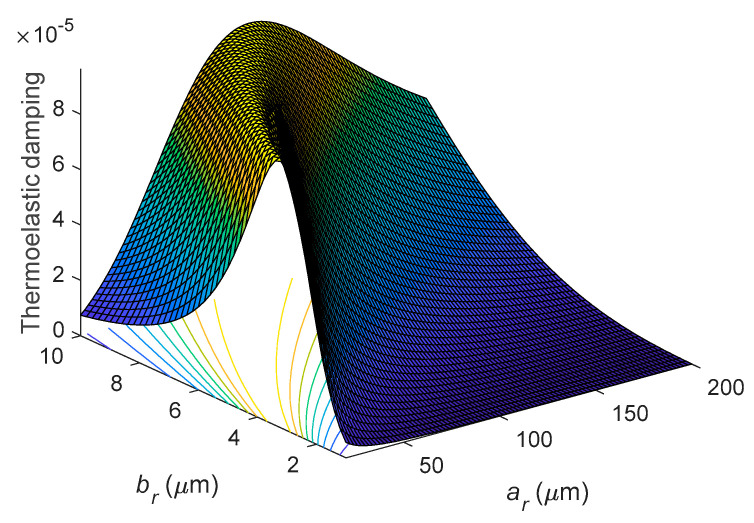
Variation of thermoelastic damping with the mean radius *a_r_* and the radial thickness *b_r_* at *n* = 2.

Figure 9 shows a 3D plot of TED for different *a_r_* and *b_r_* at *n* = 2. As shown in Figure 9, the peak value has an upper limit of approximately 9.64 × 10^−5^ for different *a_r_* and *b_r_*. As also shown in the figure, corresponding to the peak value, the larger *b_r_* is, the larger *a_r_* is.

### 3.3. Characteristics of Entropy Generation

Entropy generation is an important physical quantity in the research of thermoelastic dissipation. In this section, we will study the entropy generation behavior of ring resonators under different vibrating modes and geometry structures. According to Equation (20), the entropy generation cannot be obtained without the value of *U*_0_. For simplification, this section gives a normalized entropy generation which is a function of vibrating modes and structure dimensions, expressed as

(29) $$\Delta S_{\mathrm{normalized}}=\frac{\Delta S}{U_{0}^{2}}=\frac{A_{r}}{\xi^{2}}\cdot \frac{\Delta_{E}Ec_{r}\pi^{2}{\left(n^{2}+1\right)}^{2}b_{r}^{3}}{2T_{0}a_{r}^{4}}$$

**Figure 10:**
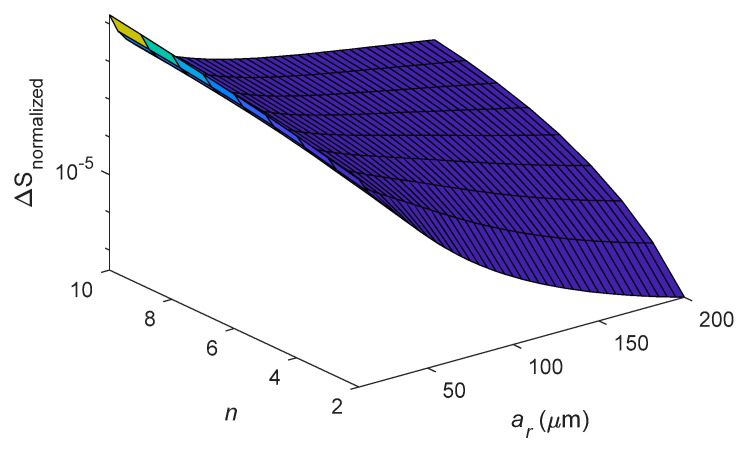
Dependence of entropy generation on varying *a_r_* and mode number *n* for the case of *b_r_* = *c_r_* = 5 μm.

**Figure 11:**
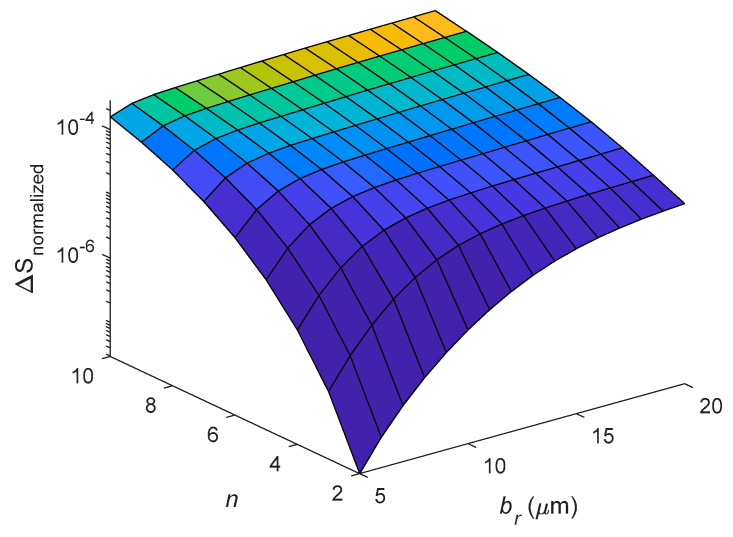
Dependence of entropy generation on varying *b_r_* and mode number *n* for constant *c_r_* = 5 μm and *a_r_* = 200 μm.

Figure 10 and Figure 11 discuss the relationship of entropy generation, mean radius *a_r_* and radial thickness *b_r_* at different eigenfrequencies. Figure 10 shows the variation of entropy generation ∆*S* with the mean radius *a_r_* for different vibrating modes *n*. The cross-section of the ring is constant, *b_r_* = *c_r_* = 5 μm. As shown in Figure 10, the entropy generation decreases with increasing mean radius *a_r_* for constant *n*. However, the entropy generation increases with increasing *n* for constant mean radius *a_r_*. Figure 11 shows the variation of entropy generation with *b_r_* and *n* for constant *c_r_* = 5 μm and *a_r_* = 200 μm. As shown in Figure 11, the entropy generation increases with increasing radial thickness *b_r_* for constant *n*. Moreover, the entropy generation is monotonically increasing with the mode number *n* for constant *b*.

**Figure 12:**
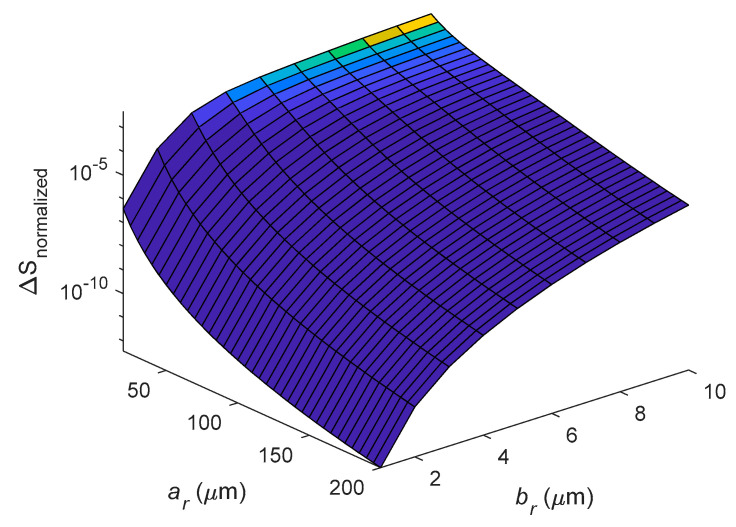
Dependence of entropy generation on varying *a_r_* and *b_r_* for the case of *n* = 2 and *b_r_* = *c_r_*.

Figure 12 shows variation of entropy generation with *a_r_* and *b_r_* under the first in-plane mode *n* = 2 with square cross-section *b_r_* = *c_r_*. Here we note that the entropy generation of a ring is proportional to the size of *b_r_* but is inversely proportional to the size of *a_r_*. Clearly, the entropy generation is more sensitive to *b_r_* than to *a_r_*.

## 4. Conclusions

This paper studied the entropy generation of a ring resonator vibrating under in-plane modes and presents an analytical model of thermoelastic damping based on entropy theory. The total entropy generation of a ring resonator during a cycle was obtained by using the complex temperature field. The present model of thermoelastic damping was verified by comparisons with experimental data, FEM simulation and the LR model. It was found that the predictions of the present model are very close to LR results with a difference less than 5%. The geometry and frequency effects on entropy generation were investigated. The peak value of thermoelastic damping wa constant and did not change with the structure dimensions of the ring. The entropy generation of a ring resonator mainly depended on the vibrating modes, structure dimensions and exciting force.

## Figures and Tables

**Table 1:** Comparison of the Zener model, LR model and the present model.

Model	Expression	Features	Simplification
Zener model	$Q_{\mathrm{Zener}}^{-1}=\Delta_{E}\frac{\omega \tau }{1+{\left(\omega \tau \right)}^{2}}$ *τ* = *b*^2^/(*π*^2^*χ*)	• Modal superposition temperature field • The first thermal mode is dominant. • Method of mechanical work loss or heat increment	High order thermal modes
LR model	$\frac{6\Delta_{E}}{\xi^{2}}\left(1-\frac{1}{\xi }\left(\frac{\sinh\xi +\sin\xi }{\cos\xi +\cosh\xi }\right)\right)$ $\xi =b_{r}\sqrt{\left(\omega /2\chi \right)}$	• Complex temperature field • Method of mechanical work loss	Complex resonant frequency or thermal stress
The present model	$\begin{array}{c} \frac{6\Delta_{E}}{\xi^{2}}\{{\left[1-\frac{3}{2\xi }\frac{\sin\xi +\sinh\xi }{\cos\xi +\cosh\xi }+\frac{1+\cos\xi \cosh\xi }{{\left(\cos\xi +\cosh\xi \right)}^{2}}\right]}^{2}\\ {+{\left[\frac{3}{2\xi }\frac{\sinh\xi -\sin\xi }{\cos\xi +\cosh\xi }-\frac{\sin\xi \sinh\xi }{{\left(\cos\xi +\cosh\xi \right)}^{2}}\right]}^{2}\}}^{\frac{1}{2}} \end{array}$	• Complex temperature field • Method of heat increment related to entropy generation	Temperature

**Table 2:** Material properties of polysilicon at 300 K [30].

Parameters	Polysilicon
Young’s modulus, *E* (GPa)	157
Poisson’s ratio, *υ*	0.22
Density, *ρ* (kg m^−3^)	2330
Thermal conductivity, *κ* (W m^−1^ K^−1^)	90
Specific heat, *C_p_* (J kg^−1^ K^−1^)	699
Thermal expansion coefficient, *α* (K^−1^)	2.6 × 10^−6^

**Table 3:** Comparison of measured and predicted thermoelastic damping in rings with different dimensions.

*a_r_* (mm)	*b_r_* (μm)	Mode (*n* = 2) (kHz)	*Q* ^−1^		% Error
			Measured [18]	The Present Model	
**3**	**120**	**13.8**	9.5238 × 10^−5^	9.2843 × 10^−5^	−2.51
3	117	13.49	1.0000 × 10^−5^	9.5178 × 10^−5^	−4.82
2	50	12.97	4.1667 × 10^−5^	4.5396 × 10^−5^	8.95
2	52	13.49	4.5455 × 10^−5^	5.0340 × 10^−5^	10.75
2	38	9.85	2.0833 × 10^−5^	2.0830 × 10^−5^	−0.01

**Table 4:** Material properties of silicon for different temperatures.

Item	Value				
Temperature (K)	240	258	298	320	348
*α* (×10^−6^ K^−1^)	1.99	2.24	2.60	2.85	3.06
*C_v_* (×10^−6^ J m^−3^ K^−1^)	1.51	1.52	1.64	1.68	1.73
*χ* (×10^−5^ m^2^ s^−1^)	14.3	11.7	8.60	7.92	6.97

## Appendix

Using the expression Δ*_E_* = *Eα*^2^*T*_0_/*C_v_* and integrating Equation (19) over the whole volume of the ring, the entropy generation per cycle for the entire structure is obtained, given by

(A1) $$\begin{array}{cc} \Delta S & =\int_{V}\Delta sdV\\ & ={∭}_{V}\frac{\pi \kappa }{\omega T_{0}^{2}}{\left\{\frac{\Delta_{E}}{\alpha }\frac{1}{a_{r}^{2}}\left(\frac{\partial^{2}U\left(\varphi \right)}{\partial \varphi^{2}}+U\left(\varphi \right)\right)\right\}}^{2}{\left(1-\frac{\cos\left(px\right)}{\cos\left(\frac{pb_{r}}{2}\right)}\right)}^{2}dV\\ & =\frac{\pi \kappa \Delta_{E}^{2}}{\omega T_{0}^{2}\alpha^{2}a_{r}^{4}}\int_{-b_{r}/2}^{b_{r}/2}{\left(1-\frac{\cos\left(px\right)}{\cos\left(\frac{pb_{r}}{2}\right)}\right)}^{2}dx\int_{0}^{2\pi }{\left[\frac{\partial^{2}U\left(\varphi \right)}{\partial \varphi^{2}}+U\left(\varphi \right)\right]}^{2}d\varphi \int_{-c_{r}/2}^{c_{r}/2}dZ\\ & =\frac{\pi \kappa b_{r}c_{r}\Delta_{E}^{2}}{\omega T_{0}^{2}\alpha^{2}a_{r}^{4}}\underset{A_{r}}{\underset{︸}{\left[1+\frac{1}{1+\cos\left(pb_{r}\right)}-\frac{3}{pb_{r}}\tan\left(\frac{pb_{r}}{2}\right)\right]}}\pi {\left(n^{2}+1\right)}^{2}U_{0}^{2}\\ & =\frac{A_{r}}{\xi^{2}}\cdot \frac{\Delta EEc_{r}\pi^{2}{\left(n^{2}+1\right)}^{2}U_{0}^{2}b_{r}^{3}}{2T_{0}a_{r}^{4}} \end{array}$$

For small vibration of in-plane mode of a ring, consider only the strain and stress along circumferential direction to calculate the total energy stored per cycle of entire structure. Using Equations (7) and (8), the maximum energy stored is given by

(A2) $$\begin{array}{cc} W_{\mathrm{stored}} & =\frac{1}{2}\int_{V}{\hat{\sigma }}_{\varphi }{\hat{\varepsilon }}_{\varphi }dV\\ & =\frac{E}{2}\int_{V}{\left[\frac{x}{a_{r}^{2}}\left(\frac{\partial^{2}U\left(\varphi \right)}{\partial \varphi^{2}}+U\left(\varphi \right)\right)\right]}^{2}dV\\ & =\frac{E}{2a_{r}^{4}}\int_{-b_{r}/2}^{b_{r}/2}x^{2}dx\int_{0}^{2\pi }{\left[\frac{\partial^{2}U\left(\varphi \right)}{\partial \varphi^{2}}+U\left(\varphi \right)\right]}^{2}d\varphi \int_{-c_{r}/2}^{c_{r}/2}dZ\\ & =\frac{Ec_{r}\pi {\left(n^{2}+1\right)}^{2}U_{0}^{2}b_{r}^{3}}{24a_{r}^{4}} \end{array}$$
